# Abyssal fauna of the UK-1 polymetallic nodule exploration area, Clarion-Clipperton Zone, central Pacific Ocean: Cnidaria

**DOI:** 10.3897/BDJ.4.e9277

**Published:** 2016-06-30

**Authors:** Thomas G Dahlgren, Helena Wiklund, Muriel Rabone, Diva J Amon, Chiho Ikebe, Les Watling, Craig R Smith, Adrian G Glover

**Affiliations:** ‡ Uni Research, Bergen, Norway; § Department of Marine Sciences, University of Gothenburg, Gothenburg, Sweden; | Natural History Museum, London, United Kingdom; ¶ University of Hawaii at Manoa, Honolulu, United States of America

**Keywords:** DNA barcode, deep sea, coral, sea anemony, sea jelly, baseline, survey, mining, CCZ, megafauna

## Abstract

**Background:**

We present data from a DNA taxonomy register of the abyssal Cnidaria collected as part of the Abyssal Baseline (ABYSSLINE) environmental survey cruise ‘AB01’ to the UK Seabed Resources Ltd (UKSRL) polymetallic-nodule exploration area ‘UK-1’ in the eastern Clarion-Clipperton Zone (CCZ), central Pacific Ocean abyssal plain. This is the second paper in a series to provide regional taxonomic data for a region that is undergoing intense deep-sea mineral exploration for high-grade polymetallic nodules. Data were collected from the UK-1 exploration area following the methods described in Glover et al. (2015b).

**New information:**

Morphological and genetic data are presented for 10 species and 18 records identified by a combination of morphological and genetic data, including molecular phylogenetic analyses. These included 2 primnoid octocorals, 2 isidid octocorals, 1 anemone, 4 hydroids (including 2 pelagic siphonophores accidentally caught) and a scyphozoan jellyfish (in the benthic stage of the life cycle). Two taxa matched previously published genetic sequences (pelagic siphonophores), two taxa matched published morphological descriptions (abyssal primnoids described from the same locality in 2015) and the remaining 6 taxa are potentially new species, for which we make the raw data, imagery and vouchers available for future taxonomic study. We have used a precautionary approach in taxon assignments to avoid over-estimating species ranges. The Clarion-Clipperton Zone is a region undergoing intense exploration for potential deep-sea mineral extraction. We present these data to facilitate future taxonomic and environmental impact study by making both data and voucher materials available through curated and accessible biological collections. For some of the specimens we also provide image data collected at the seabed by ROV, wich may facilitate more accurate taxon designation in coming ROV or AUV surveys.

## Introduction

We present data from a DNA taxonomy register of the abyssal Cnidaria collected as part of the Abyssal Baseline (ABYSSLINE) environmental survey cruise ‘AB01’ to the UK Seabed Resources Ltd (UKSRL) polymetallic-nodule exploration area ‘UK-1’ in the eastern Clarion-Clipperton Zone (CCZ), central Pacific Ocean (Smith et al. 2013).

This is the second paper in a series to provide taxonomic data for a region that is undergoing intense deep-sea mineral exploration for high-grade polymetallic nodules regulated by Sponsoring States (here the United Kingdom Government) and the International Seabed Authority (ISA 2014, Glover and Smith 2003, Glover et al. 2015b, Wedding et al. 2015). As with the previous Echinodermata paper (Glover et al. 2016) this Cnidaria contribution is not yet a comprehensive faunal guide to the region, but a data paper that may be updated with new additions following future collections and analyses. New versions will contain all the data contained in the previous version, plus additional descriptions and records from future research cruises.

The abyssal zone of the world’s oceans has been defined as the seafloor between depths of 3000m and 6000m, a bathymetric zone that encompasses 54% of the geographic surface of the planet (Smith et al. 2008). Cnidarians form a morphologically and functionally diverse group in this region. Current online data sources list 616 cnidarian species recorded at abyssal depths from between 3000m and 6000m (OBIS 2016) out of a total of 1,922 cnidarian species recorded from depths greater than 500m (Glover et al. 2015a).

**Figure 1.**
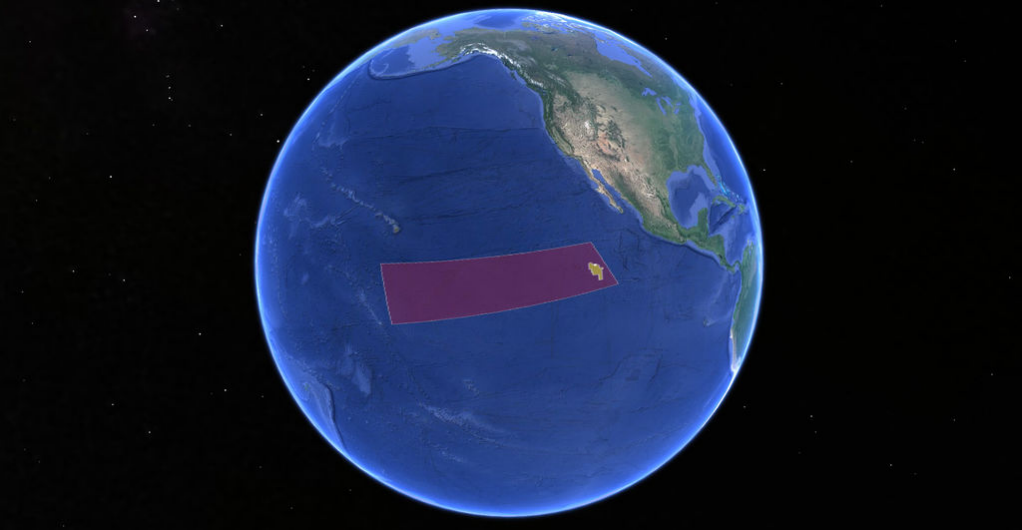
The Clarion-Clipperton Zone, central Pacific Ocean (purple box) is a 6 milllion sq km region at the time of writing containing only 8 online databased records of cnidarian species (OBIS 2016). The UK Seabed Resources Ltd ‘UK-1’ polymetallic nodule exploration area is highlighted (yellow box). Image via Google Earth, IBCAO, Landsat, Data from SIO, NOAA, U.S Navy, NGA, GEBCO.

The Clarion-Clipperton Zone (hereafter, CCZ) is so called as it lies mainly between the Clarion and Clipperton Fracture Zones, topographical highs that extend longitudinally across almost the entire eastern Pacific. There is no strict definition of the region, but it has come to be regarded as the area between these fracture zones that lies within international waters and encompasses the main areas of commercial interest for polymetallic-nodule mining. Areas licensed for mining by the International Seabed Authority (ISA), as well as mining reserve areas and areas protected from mining by the ISA (ISA 2014, Wedding et al. 2013) extend from 115°W (the easternmost extent of the UK-1 exploration area) to approximately 158°W, and from 22°N to 2.5°S (Fig. 1). This is an area of 6 million sq km, approximately 1.7% of the ocean’s surface.

Within the entire 6 million sq km CCZ, as defined above, current online data sources prior to this publication list three known benthic species of cnidarians from 8 records (OBIS 2016). This is obviously the result of lack of sampling and/or taxonomy given that an abundant and diverse cnidarian fauna is suspected in the region from photographic and video survey (e.g. Amon et al. 2016, Foell and Pawson 1986). The goal of the DNA taxonomy part of the ABYSSLINE program is to begin filling these gaps in our knowledge and to make data publically available, which will eventually allow for a complete taxonomic synthesis of the CCZ supported by openly-available molecular and morphological data. Here we provide version 1.0 of the Cnidaria taxonomic synthesis from the ABYSSLINE program, consisting of taxon records, high-resolution imagery, genetic data from multiple markers and phylogenetic analysis from the first research cruise (AB01) aboard the RV *Melville* in October 2013. In this paper we report seven species of benthic cnidarians from the CCZ with the mention of an additional three pelagic ones, and a total of 18 records. Benthic surveys such as ABYSSLINE often recover miscellaneous pelagic taxa (e.g recovered on wires or equipment) and one of the goals of the DNA taxonomy part of this project was to ensure that these useful data records are made available, particularly given the lack of data on pelagic species in the open ocean. This open data publication is the second of equivalent similar data publications including also the Annelida, Mollusca, Bryozoa, Echinodermata, Porifera and other taxa forming a suite of taxonomic syntheses of biodiversity in the region, supported by a contract between the company UK Seabed Resources Ltd and the Natural History Museum, London, Uni Research, Bergen, and the University of Hawaii at Manoa (Glover et al. 2015b, Glover et al. 2016).

## Materials and methods

It is widely accepted that knowledge of baseline biodiversity and biogeography in the CCZ is severely hampered by a lack of modern DNA-supported taxonomic studies (International Seabed Authority 2014). With this in mind, four fundamental principles underpin our methodological pipeline: (1) A sampling design pipeline with consideration to the spatial scale of the required data, the differing biases in sampling gear and the requirement for at-sea taxonomic study, (2) A field pipeline with consideration to the successful collection of high-quality specimens using live-sorting in a 'cold-chain' from depths of 4000-5000m in the central tropical Pacific, (3) A laboratory pipeline with consideration to the needs to collect both DNA sequences and morphological data in a timely and cost-effective manner suited to the immediate needs of the science community and, (4) A data and sample management pipeline that includes the publication of results with consideration to the accessibility of data and materials. Our complete methodology for DNA taxonomy in the CCZ, including deployment protocols for the various sampling gears, methods for live-sorting and microscope photography at sea and details of sample and data curation are provided in a separate open-access publication (Glover et al. 2015b). Details of the taxonomical methods, including primers used for DNA taxonomy, are also given in the CCZ Echinodermata paper (Glover et al. 2016).

### Field Pipeline

**Figure 2.**
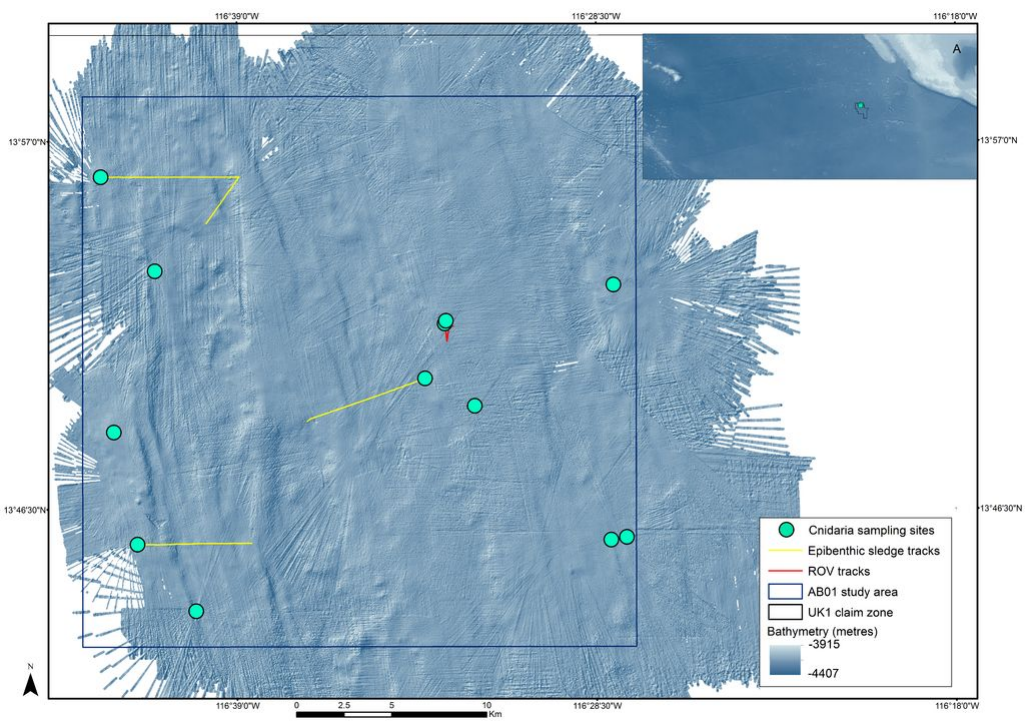
'UK-1 Stratum A' ABYSSLINE biological baseline survey box sited within the UK-1 polymetallic nodule exploration area. Stratum A is a 30x30km survey box in the northern sector of the 58,000 km^2^ exploration area. Cnidaria sample localities are indicated by green circles from the AB01 RV *Melville* survey cruise, October 2013. Inset map A: the site location within the central Pacific, with GEBCO 2014 bathymetry (global 30 arc-second interval grid data set). Seafloor bathymetry from the RV Melville ABYSSLINE cruise is shown in the main map.

The ABYSSLINE environmental baseline survey includes three 30x30 km survey boxes (strata), distributed across the UK-1 area, and an additional reference sites outside of the UK-1 area (Glover et al. 2015b, Smith et al. 2013). Within each survey stratum, sample sites for a variety of benthic sampling gears are selected randomly – a randomized, stratified sampling design that assumes no *a priori* knowledge of the benthic environment (Smith et al. 2013). The UK-1 strata are being sampled in a series of oceanographic cruises during the course of the project, which commenced in July 2013, with the first cruise (AB01) taking place in October 2013 aboard the RV *Melville*. During this cruise, the first stratum was comprehensively mapped with multibeam bathymetry and sampled for a range of biological, environmental and geophysical parameters (Fig. 2, Smith et al. 2013).

**Figure 3.**
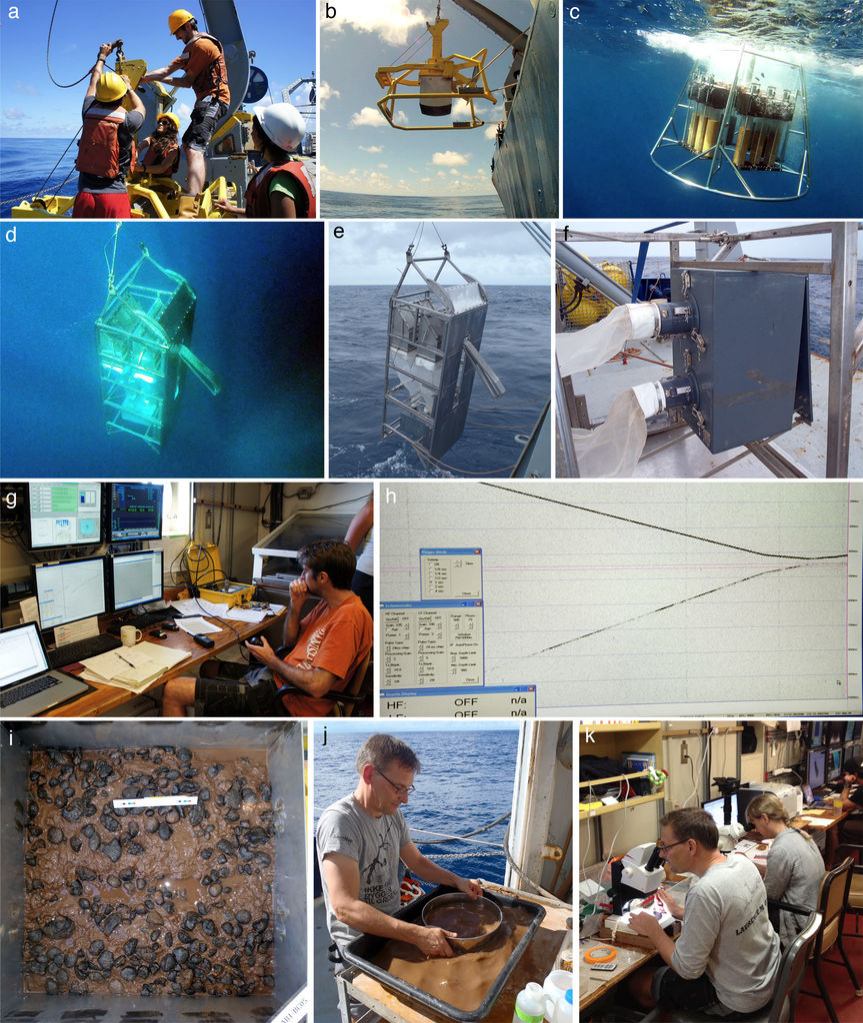
ABYSSLINE UK-1 polymetallic nodule exploration area field pipeline for DNA taxonomy. ABYSSLINE AB01 cruise sampling aboard RV *Melville* in October 2013. (a) Preparing Box Core (BC) for deployment, (b) BC entering the water, (c) Megacore entering the water, (d-f) Epibenthic Sledge shown on recovery in water and cod-end where samples are taken, (g) controlling BC deployment on seafloor, (h) echosounder trace showing BC approaching seabed reflection, (i) successful BC surface after recovery, 50cm x 50cm, (j) carefully sifting mud in chilled filtered seawater (approx. temp 5-7°C) to remove live animals in undamaged state, (k) live-sorting aboard ship, taking samples for DNA and photomicrographs of specimens down to <1mm in size. All images by Glover, Dahlgren & Wiklund. A more comprehensive description of our methods is provided in Glover et al. 2015b.

A comprehensive description of our DNA taxonomy pipeline is provided in Glover et al. 2015b. In summary, deep-sea benthic specimens from the UK-1 Stratum A were collected using a range of oceanographic sampling gears including box core (BC), epibenthic sledge (EBS), remotely operated vehicle (ROV) and megacore (MC) (Figs 2, 3). Geographic data from sampling activities were recorded on a central GIS database. Live-sorting of sediment and specimen samples was carried out aboard the RV *Melville* under the ‘cold-chain’ pipeline, in which material was immediately transferred and maintained in chilled, filtered seawater held at 2-4°C (Fig. 3​). Specimens were preliminarily identified at sea and imaged live using stereomicroscopes with attached digital cameras and strobe lighting. The specimens were then transferred to individual microtube vials containing an aqueous solution of 80% non-denatured ethanol, numbered and barcoded into a database and kept chilled until return to the Natural History Museum (NHM), London. Larger, megafaunal-sized, animals were sub-sampled for DNA (with the tissue and DNA sample being taken to NHM, London) with the remaining intact specimen preserved in 10% formalin solution and taken to the University of Hawaii, Honolulu, USA for further study.

### Laboratory Pipeline

Extraction of DNA was done with DNeasy Blood and Tissue Kit (Qiagen) using a Hamilton Microlab STAR Robotic Workstation. About 1800 bp of 18S, 450 bp of 16S, and 650 bp of cytochrome c oxidase subunit I (COI) were amplified using primers listed in Table 1. PCR mixtures contained 1 μl of each primer (10μM), 2 μl template DNA and 21 μl of Red Taq DNA Polymerase 1.1X MasterMix (VWR) in a mixture of total 25 μl. The PCR amplification profile consisted of initial denaturation at 95°C for 5 min, 35 cycles of denaturation at 94°C for 45 s, annealing at 55°C for 45 s, extension at 72°C for 2 min, and a final extension at 72°C for 10 min. PCR products were purified using Millipore Multiscreen 96-well PCR Purification System, and sequencing was performed on an ABI 3730XL DNA Analyser (Applied Biosystems) at The Natural History Museum Sequencing Facility, using the same primers as in the PCR reactions plus two internal primers for 18S (Table 1).

Overlapping sequence fragments were merged into consensus sequences using Geneious R6 (www.geneious.com, Kearse et al. (2012) and aligned using MAFFT (Katoh 2002) for 18S and 16S, and MUSCLE (Edgar 2004) for COI, both programs used as plugins in Geneious, with default settings. Bayesian phylogenetic analyses (BA) were conducted with MrBayes 3.1.2 (Ronquist and Huelsenbeck 2003). Analyses were run for 10-20 million generations, of which 2.5-5 million generations were discarded as burn-in.

### Data Pipeline

**Figure 4.**
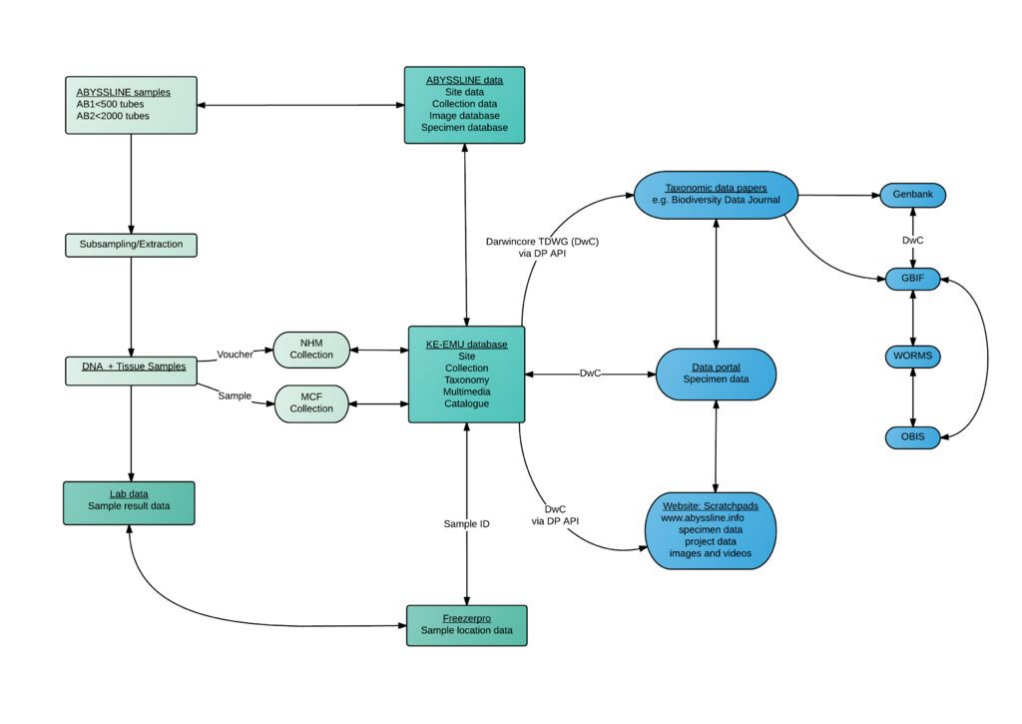
Data and sample management workflow on the ABYSSLINE DNA taxonomy project. Processes relating to a) physical samples are shown in grey, b) institution level data in dark green and c) externally-available data in blue.

The field and laboratory pipelines created a series of databases and sample sets that were then integrated into a data-management pipeline (Fig. 4). This includes the transfer and management of data and samples between a central collections database, a molecular collections database, an online scratchpad (website for faunal data) and external repositories (e.g GenBank, WoRMS, OBIS, GBIF) through a DarwinCore archive. This provides a robust data framework to support DNA taxonomy, in which openly-available data and voucher material is key to quality data standards. A further elaboration of the data pipeline is published in Glover et al. (2015b)

### Taxonomic Assignments

All future studies of biogeographic and bathymetric ranges, gene-flow, extinction risks, natural history, reproductive ecology, functional ecology and geochemical interactions of CCZ species are dependent on accurate identifications faciliated by taxonomy. This taxonomy, presented here, is itself dependent on a sound theoretical underpinning – a species concept - coupled with the availability of both raw data and voucher samples. Here we use a phylogenetic species concept, *sensu* Donoghue 1985 with species determined by DNA-based phylogenetic analysis and the recognition of distinct monophyletic groups as species. For those taxa where the typical morphological data that allows determination of species are missing, we provide the lowest-level taxonomic name possible, but determination to species with genetic data. For species similar to a morphologically well-defined species name where we lack comparable genetic data from type material or from the type locality, or when genetic data previously published in Genbank is incompatible with ours, we use the open nomenclature expression ”*cf.*".

All DNA vouchers (including archived frozen tissue) and genetic data are accessible through the Natural History Museum, London, together with the morphological data presented in this paper. Additional morphological material is held at both NHM London and the University of Hawaii (detailed in Table 2). As such our species hypotheses are easily open to further evaluation and iterative improvement, e.g. full descriptions for new taxa with improved data from future cruises. A localized identification field guide to the CCZ fauna will be the subject of a future publication as more species are described, but for the present we recommend DNA-based identification (barcoding) of our species coupled with morphological comparisons made possible through this publication.

## Data resources

The following sections detail the phylogenetic analysis and data resources that underpins the species hypotheses presented in the taxon treatments. A full list of all taxa including Natural History Museum Accession Numbers, NHM Molecular Collection Facility (NHM-MCF) FreezerPro numbers and NCBI GenBank Accession numbers is provided in Table 2.

### Phylogenetic analyses

All specimens in Table 2 with the exception of NHM_083 and NHM_321 were sequenced, searched via Basic Local Alignment Search Tool (BLAST) for comparison on NCBI Genbank and subsequently analysed in MrBayes for phylogenetic relationship studies.

**Figure 5.**
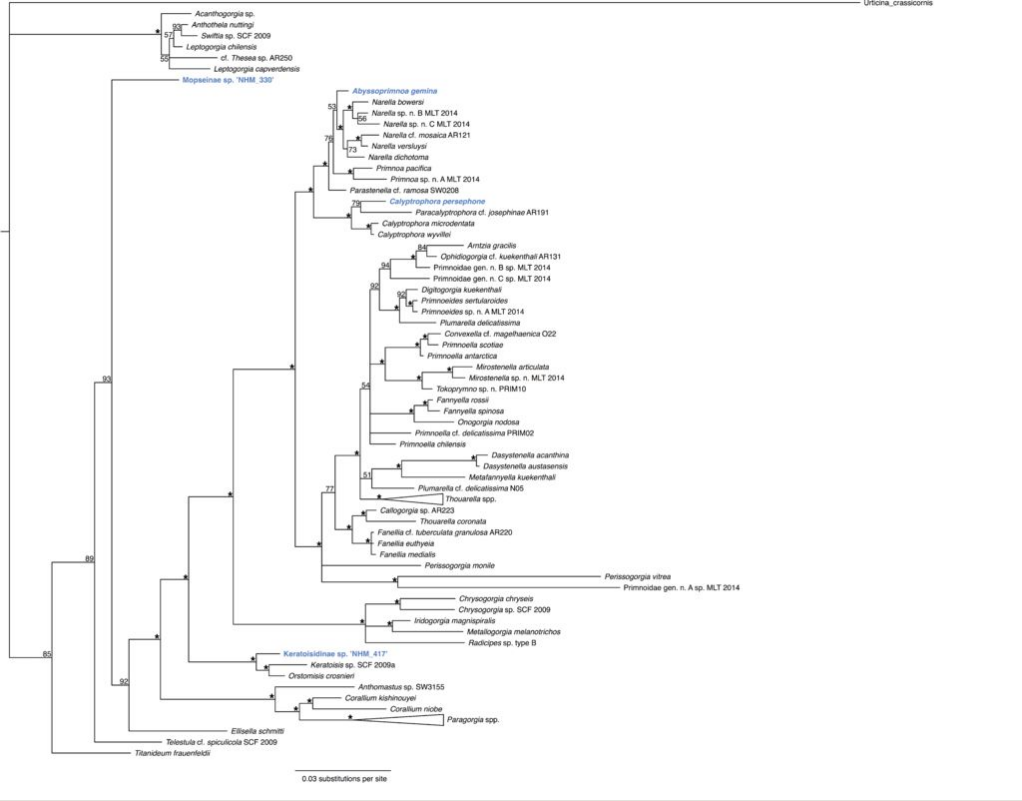
Phylogenetic analysis of the octocorals. 50% majority rule consensus tree from the Bayesian analyses, combining the two genes 18S and 16S, and using in total 83 taxa (Suppl. material 1). Some of the clades are collapsed in order to make the tree smaller and easier to read.

Although phylogenetic trees were produced for all species, we only present one of the trees here, the octocorals (Fig. 5). In all other analyses, the specimens were too distant from each other to incorporate in one tree per group, and their affiliations are instead explained in the text below.

Phylogenetic analysis of the octocorals (Fig. 5) reveals the presence of four distinct lineages of ABYSSLINE specimens which we interpret as the four species described below based on their genetic data. Among Hydrozoa, two of the sequenced species had a match on Genbank; *Abylopsis eschscholtzii* NHM_477 (matching with accession number GQ119937) and *Stephanomia amphytridis* NHM_249 (matching with GQ120047), and subsequent alignment view and tree building (trees not showed) confirmed the identifications. For some of the other taxa, trees were constructed in order to narrow down the taxonomic identity, and thus the hydrozoan NHM_398 was shown to fall within the family Corymorphidae, while the scyphozoan NHM_353 fell close to *Nausithoe atlantica*. As only COI from one species of Nausithoe was available, it is impossible to prove that NHM_353 actually belongs to that genus. However, with an uncorrected ‘p’ and K2P value of 0.04 between *Nausithoe atlantica* and NHM_353, which is comparable to values of 0.06 between *Atolla vanhoeffeni* and *Atolla wyvillei* (also Coronatae, as is *Nausithoe*), we suggest *Nausithoe* sp. for NHM_353 for now. The siphonophore NHM_339 was impossible to identify to any taxonomic level below order. The hexacoral NHM_416 was identified by morphology to the family Hormathiidae, and while the molecular result was inconclusive it was not contradictory, and future studies might resolve the generic affiliation.

## Taxon treatments

### Abyssoprimnoa gemina

Cairns, 2015

#### Materials

- **Type status:** Other material. **Occurrence:** catalogNumber: 6e976c27-c70c-434a-8be3-f6155e36567f; recordNumber: NHM_341; recordedBy: Adrian Glover, Helena Wiklund, Thomas Dahlgren, Maggie Georgieva; individualCount: 1; preparations: tissue voucher stored in 80% non-denatured ethanol aqueous solution and DNA voucher stored in elution buffer; otherCatalogNumbers: 5594618; associatedSequences: http://ncbi.nlm.nih.gov/nucleotide/KX384618 | KX384626; **Taxon:** taxonConceptID: Abyssoprimnoa gemina; scientificName: Abyssoprimnoa gemina; kingdom: Animalia; phylum: Cnidaria; class: Anthozoa; order: Alcyonacea; family: Primnoidae; genus: Abyssoprimnoa; specificEpithet: gemina; scientificNameAuthorship: Cairns, 2015; **Location:** waterBody: Pacific; stateProvince: Clarion Clipperton Zone; locality: UK Seabed Resources Ltd exploration claim UK-1; verbatimLocality: UK-1 Stratum A; maximumDepthInMeters: 4111; locationRemarks: RV Melville Cruise MV1313; decimalLatitude: 13.761666666667; decimalLongitude: -116.46033333333; geodeticDatum: WGS84; **Identification:** identifiedBy: Stephen Cairns, Adrian Glover, Helena Wiklund, Thomas Dahlgren, Diva Amon; dateIdentified: 2016-03-01; identificationRemarks: identified by DNA and morphology; **Event:** samplingProtocol: Bowers & Connelly Megacore; eventDate: 2013-10-18; eventTime: 15:54; habitat: Abyssal plain; fieldNumber: MC08; **Record Level:** language: en; institutionCode: NHMUK; collectionCode: ZOO; datasetName: ABYSSLINE; basisOfRecord: PreservedSpecimen

#### Description

**Figure 6.**
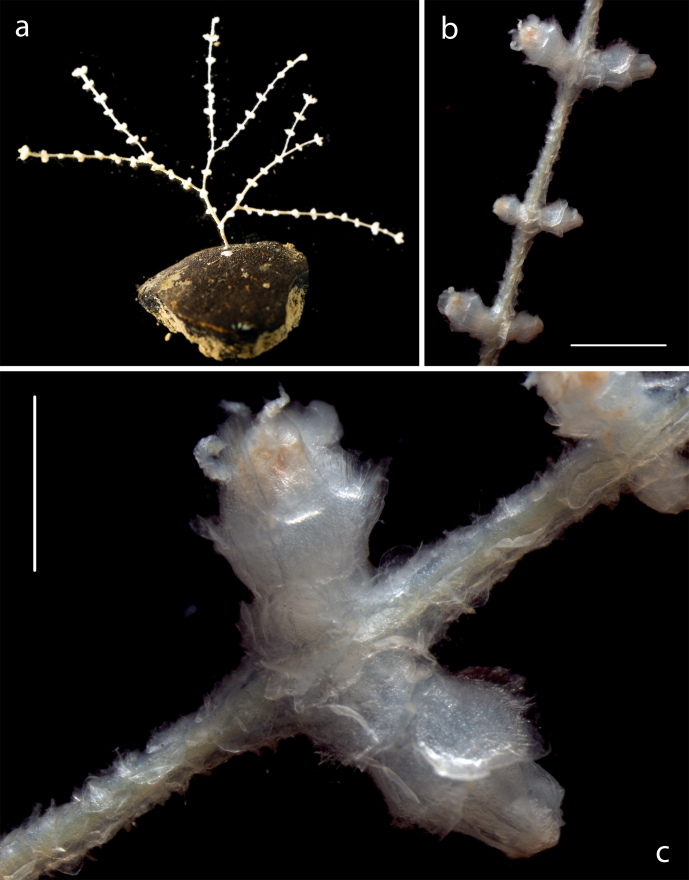
*Abyssoprimnoa gemina* Cairns, 2015. (a) Specimen NHM_341 attached to nodule. (b) Detail from same specimen. (c) Polyp from same specimen. Scale bars (b) 2 mm, (c) 1 mm. Image attribution Glover, Dahlgren & Wiklund 2016.

Small uniplanar dichotomously branched colonies, having paired globose polyps (Fig. 6).

Genetic data for this taxon with new GenBank accession numbers are provided in Table 2.

#### Diagnosis

Species described by Stephen Cairns based on this specimen (NHM_341) from UK-1 claim area and additional material collected in the German claim area (Cairns 2015). No genetic matches at Genbank but forms a cluster with *Narella* and *Primnoa* sequences published.

### Calyptrophora persephone

Cairns, 2015

#### Materials

- **Type status:** Other material. **Occurrence:** catalogNumber: dc143dd0-a366-479a-9e23-ca28fe79761b; recordNumber: NHM_462; recordedBy: Adrian Glover, Helena Wiklund, Thomas Dahlgren, Maggie Georgieva; individualCount: 1; preparations: tissue voucher stored in 80% non-denatured ethanol aqueous solution and DNA voucher stored in elution buffer; otherCatalogNumbers: 5594705; associatedSequences: http://ncbi.nlm.nih.gov/nucleotide/KX384619 | KX384627; **Taxon:** taxonConceptID: Calyptrophora persephone; scientificName: Calyptrophora persephone; kingdom: Animalia; phylum: Cnidaria; class: Anthozoa; order: Alcyonacea; family: Primnoidae; genus: Calyptrophora; specificEpithet: persephone; scientificNameAuthorship: Cairns, 2015; **Location:** waterBody: Pacific; stateProvince: Clarion Clipperton Zone; locality: UK Seabed Resources Ltd exploration claim UK-1; verbatimLocality: UK-1 Stratum A; maximumDepthInMeters: 4160; locationRemarks: RV Melville Cruise MV1313; decimalLatitude: 13.726616666667; decimalLongitude: -116.67; geodeticDatum: WGS84; **Identification:** identifiedBy: Stephen Cairns, Adrian Glover, Helena Wiklund, Thomas Dahlgren, Diva Amon; dateIdentified: 2016-03-01; identificationRemarks: identified by DNA and morphology; **Event:** samplingProtocol: USNEL Box Core; eventDate: 2013-10-22; eventTime: 07:25; habitat: Abyssal plain; fieldNumber: BC14; fieldNotes: Collected from 0-2 cm layer of box core using a 300 micron sieve; **Record Level:** language: en; institutionCode: NHMUK; collectionCode: ZOO; datasetName: ABYSSLINE; basisOfRecord: PreservedSpecimen

#### Description

**Figure 7.**
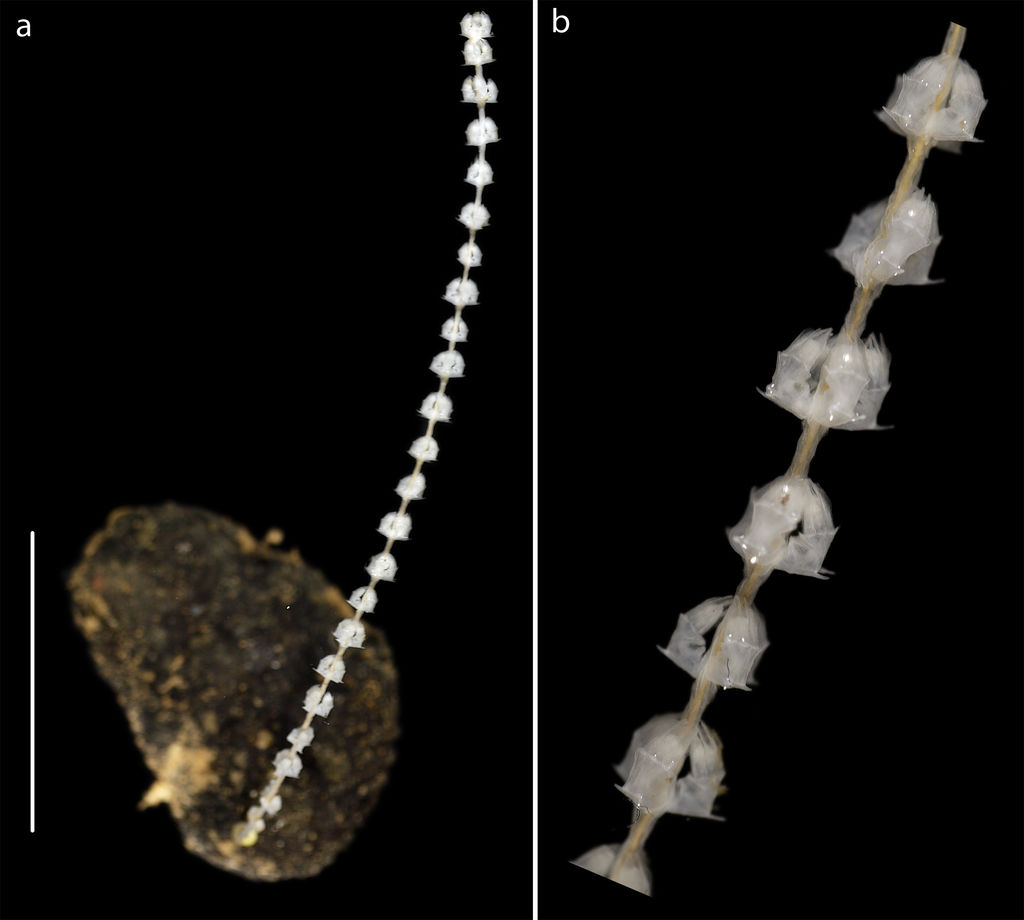
*Calyptrophora persephone* Cairns, 2015. (a) Specimen NHM_462 attached to nodule, (b) Detail, same specimen. Scale bar 30 mm. Image attribution Glover, Dahlgren & Wiklund 2016.

Unbranched upright colonies with worls of three to four polyps facing upward on stem Fig. 7.

Genetic data for this taxa with new Genbank accession numbers are provided in Table 2.

#### Diagnosis

Species described by Stephen Cairns based on this specimen (NHM_462) and additional material collected in the German claim area (Cairns 2015). No genetic matches at Genbank but forms a cluster with other *Calyptrophora* sequences published

### Mopseinae sp. 'NHM_330'

#### Materials

- **Type status:** Other material. **Occurrence:** catalogNumber: 52fc66a5-d396-49be-a210-4ac58a164aec; recordNumber: NHM_002; recordedBy: Adrian Glover, Helena Wiklund, Thomas Dahlgren, Maggie Georgieva; individualCount: 1; preparations: tissue voucher stored in 80% non-denatured ethanol aqueous solution and DNA voucher stored in elution buffer; otherCatalogNumbers: 5595039; associatedSequences: http://ncbi.nlm.nih.gov/nucleotide/KX384629; **Taxon:** taxonConceptID: Mopseinae sp. (NHM_330); scientificName: Mopseinae; kingdom: Animalia; phylum: Cnidaria; class: Anthozoa; order: Alcyonacea; family: Isididae; scientificNameAuthorship: Lamouroux, 1812; **Location:** waterBody: Pacific; stateProvince: Clarion Clipperton Zone; locality: UK Seabed Resources Ltd exploration claim UK-1; verbatimLocality: UK-1 Stratum A; maximumDepthInMeters: 4171; locationRemarks: RV Melville Cruise MV1313; decimalLatitude: 13.881666666667; decimalLongitude: -116.46666666667; geodeticDatum: WGS84; **Identification:** identifiedBy: Les Watling, Adrian Glover, Helena Wiklund, Thomas Dahlgren, Diva Amon; dateIdentified: 2016-03-01; identificationRemarks: identified by DNA and morphology; **Event:** samplingProtocol: USNEL Box Core; eventDate: 2013-10-08; eventTime: 17:15; habitat: Abyssal plain; fieldNumber: BC03; fieldNotes: Collected from 0-2 cm layer of box core using a 300 micron sieve; **Record Level:** language: en; institutionCode: NHMUK; collectionCode: ZOO; datasetName: ABYSSLINE; basisOfRecord: PreservedSpecimen
- **Type status:** Other material. **Occurrence:** catalogNumber: c1c12d85-274b-4ae5-bf60-72c82b70dc16; recordNumber: NHM_019; recordedBy: Adrian Glover, Helena Wiklund, Thomas Dahlgren, Maggie Georgieva; individualCount: 1; preparations: tissue voucher stored in 80% non-denatured ethanol aqueous solution and DNA voucher stored in elution buffer; otherCatalogNumbers: 5594370; associatedSequences: http://ncbi.nlm.nih.gov/nucleotide/KX384610 | KX384621 | KX384630; **Taxon:** taxonConceptID: Mopseinae sp. (NHM_330); scientificName: Mopseinae; kingdom: Animalia; phylum: Cnidaria; class: Anthozoa; order: Alcyonacea; family: Isididae; scientificNameAuthorship: Lamouroux, 1812; **Location:** waterBody: Pacific; stateProvince: Clarion Clipperton Zone; locality: UK Seabed Resources Ltd exploration claim UK-1; verbatimLocality: UK-1 Stratum A; maximumDepthInMeters: 4336; locationRemarks: RV Melville Cruise MV1313; decimalLatitude: 13.8372; decimalLongitude: -116.55843333333; geodeticDatum: WGS84; **Identification:** identifiedBy: Les Watling, Adrian Glover, Helena Wiklund, Thomas Dahlgren, Diva Amon; dateIdentified: 2016-03-01; identificationRemarks: identified by DNA and morphology; **Event:** samplingProtocol: Brenke Epibenthic Sledge; eventDate: 2013-10-09; eventTime: 10:26; habitat: Abyssal plain; fieldNumber: EB01; fieldNotes: Collected from epi net (on the epibenthic sledge); **Record Level:** language: en; institutionCode: NHMUK; collectionCode: ZOO; datasetName: ABYSSLINE; basisOfRecord: PreservedSpecimen
- **Type status:** Other material. **Occurrence:** catalogNumber: 39fb53c7-b5f4-4e9a-a8da-756129250937; recordNumber: NHM_035; recordedBy: Adrian Glover, Helena Wiklund, Thomas Dahlgren, Maggie Georgieva; individualCount: 1; preparations: tissue voucher stored in 80% non-denatured ethanol aqueous solution and DNA voucher stored in elution buffer; otherCatalogNumbers: 5594384; associatedSequences: http://ncbi.nlm.nih.gov/nucleotide/KX384611; **Taxon:** taxonConceptID: Mopseinae sp. (NHM_330); scientificName: Mopseinae; kingdom: Animalia; phylum: Cnidaria; class: Anthozoa; order: Alcyonacea; family: Isididae; scientificNameAuthorship: Lamouroux, 1812; **Location:** waterBody: Pacific; stateProvince: Clarion Clipperton Zone; locality: UK Seabed Resources Ltd exploration claim UK-1; verbatimLocality: UK-1 Stratum A; maximumDepthInMeters: 4336; locationRemarks: RV Melville Cruise MV1313; decimalLatitude: 13.8372; decimalLongitude: -116.55843333333; geodeticDatum: WGS84; **Identification:** identifiedBy: Les Watling, Adrian Glover, Helena Wiklund, Thomas Dahlgren, Diva Amon; dateIdentified: 2016-03-01; identificationRemarks: identified by DNA and morphology; **Event:** samplingProtocol: Brenke Epibenthic Sledge; eventDate: 2013-10-09; eventTime: 10:26; habitat: Abyssal plain; fieldNumber: EB01; fieldNotes: Collected from epi net (on the epibenthic sledge); **Record Level:** language: en; institutionCode: NHMUK; collectionCode: ZOO; datasetName: ABYSSLINE; basisOfRecord: PreservedSpecimen
- **Type status:** Other material. **Occurrence:** catalogNumber: fb7d366b-dcd6-448e-8fe0-7625d811483d; recordNumber: NHM_136; recordedBy: Adrian Glover, Helena Wiklund, Thomas Dahlgren, Maggie Georgieva; individualCount: 1; preparations: tissue voucher stored in 80% non-denatured ethanol aqueous solution and DNA voucher stored in elution buffer; otherCatalogNumbers: 5594458; associatedSequences: http://ncbi.nlm.nih.gov/nucleotide/KX384631; **Taxon:** taxonConceptID: Mopseinae sp. (NHM_330); scientificName: Mopseinae; kingdom: Animalia; phylum: Cnidaria; class: Anthozoa; order: Alcyonacea; family: Isididae; scientificNameAuthorship: Lamouroux, 1812; **Location:** waterBody: Pacific; stateProvince: Clarion Clipperton Zone; locality: UK Seabed Resources Ltd exploration claim UK-1; verbatimLocality: UK-1 Stratum A; maximumDepthInMeters: 4080; locationRemarks: RV Melville Cruise MV1313; decimalLatitude: 13.758333333333; decimalLongitude: -116.69851666667; geodeticDatum: WGS84; **Identification:** identifiedBy: Les Watling, Adrian Glover, Helena Wiklund, Thomas Dahlgren, Diva Amon; dateIdentified: 2016-03-01; identificationRemarks: identified by DNA and morphology; **Event:** samplingProtocol: Brenke Epibenthic Sledge; eventDate: 2013-10-11; eventTime: 10:32; habitat: Abyssal plain; fieldNumber: EB02; fieldNotes: Collected from epi net (on the epibenthic sledge); **Record Level:** language: en; institutionCode: NHMUK; collectionCode: ZOO; datasetName: ABYSSLINE; basisOfRecord: PreservedSpecimen
- **Type status:** Other material. **Occurrence:** catalogNumber: 6102fb1a-c452-47a8-9a52-ca1159c06cb0; recordNumber: NHM_154; recordedBy: Adrian Glover, Helena Wiklund, Thomas Dahlgren, Maggie Georgieva; individualCount: 1; preparations: tissue voucher stored in 80% non-denatured ethanol aqueous solution and DNA voucher stored in elution buffer; otherCatalogNumbers: 5594475; associatedSequences: http://ncbi.nlm.nih.gov/nucleotide/KX384632; **Taxon:** taxonConceptID: Mopseinae sp. (NHM_330); scientificName: Mopseinae; kingdom: Animalia; phylum: Cnidaria; class: Anthozoa; order: Alcyonacea; family: Isididae; scientificNameAuthorship: Lamouroux, 1812; **Location:** waterBody: Pacific; stateProvince: Clarion Clipperton Zone; locality: UK Seabed Resources Ltd exploration claim UK-1; verbatimLocality: UK-1 Stratum A; maximumDepthInMeters: 4080; locationRemarks: RV Melville Cruise MV1313; decimalLatitude: 13.758333333333; decimalLongitude: -116.69851666667; geodeticDatum: WGS84; **Identification:** identifiedBy: Les Watling, Adrian Glover, Helena Wiklund, Thomas Dahlgren, Diva Amon; dateIdentified: 2016-03-01; identificationRemarks: identified by DNA and morphology; **Event:** samplingProtocol: Brenke Epibenthic Sledge; eventDate: 2013-10-11; eventTime: 10:32; habitat: Abyssal plain; fieldNumber: EB02; fieldNotes: Collected from epi net (on the epibenthic sledge); **Record Level:** language: en; institutionCode: NHMUK; collectionCode: ZOO; datasetName: ABYSSLINE; basisOfRecord: PreservedSpecimen
- **Type status:** Other material. **Occurrence:** catalogNumber: d73e2414-e2ad-4c07-9e12-0864ce6ef3c1; recordNumber: NHM_203; recordedBy: Adrian Glover, Helena Wiklund, Thomas Dahlgren, Maggie Georgieva; individualCount: 1; preparations: tissue voucher stored in 80% non-denatured ethanol aqueous solution and DNA voucher stored in elution buffer; otherCatalogNumbers: 5594513; associatedSequences: http://ncbi.nlm.nih.gov/nucleotide/KX384612 | KX384633; **Taxon:** taxonConceptID: Mopseinae sp. (NHM_330); scientificName: Mopseinae; kingdom: Animalia; phylum: Cnidaria; class: Anthozoa; order: Alcyonacea; family: Isididae; scientificNameAuthorship: Lamouroux, 1812; **Location:** waterBody: Pacific; stateProvince: Clarion Clipperton Zone; locality: UK Seabed Resources Ltd exploration claim UK-1; verbatimLocality: UK-1 Stratum A; maximumDepthInMeters: 4054; locationRemarks: RV Melville Cruise MV1313; decimalLatitude: 13.824116666667; decimalLongitude: -116.53425; geodeticDatum: WGS84; **Identification:** identifiedBy: Les Watling, Adrian Glover, Helena Wiklund, Thomas Dahlgren, Diva Amon; dateIdentified: 2016-03-01; identificationRemarks: identified by DNA and morphology; **Event:** samplingProtocol: USNEL Box Core; eventDate: 2013-10-14; eventTime: 21:37; habitat: Abyssal plain; fieldNumber: BC07; fieldNotes: Collected from 0-2 cm layer of box core using a 300 micron sieve; **Record Level:** language: en; institutionCode: NHMUK; collectionCode: ZOO; datasetName: ABYSSLINE; basisOfRecord: PreservedSpecimen
- **Type status:** Other material. **Occurrence:** catalogNumber: d5fdef19-8efd-4865-bc53-725568bac8df; recordNumber: NHM_330; recordedBy: Adrian Glover, Helena Wiklund, Thomas Dahlgren, Maggie Georgieva; individualCount: 1; preparations: tissue voucher stored in 80% non-denatured ethanol aqueous solution and DNA voucher stored in elution buffer; otherCatalogNumbers: 5594610; associatedSequences: http://ncbi.nlm.nih.gov/nucleotide/KX384613 | KX384634; **Taxon:** taxonConceptID: Mopseinae sp. (NHM_330); scientificName: Mopseinae; kingdom: Animalia; phylum: Cnidaria; class: Anthozoa; order: Alcyonacea; family: Isididae; scientificNameAuthorship: Lamouroux, 1812; **Location:** waterBody: Pacific; stateProvince: Clarion Clipperton Zone; locality: UK Seabed Resources Ltd exploration claim UK-1; verbatimLocality: UK-1 Stratum A; maximumDepthInMeters: 4043; locationRemarks: RV Melville Cruise MV1313; decimalLatitude: 13.7603833333333; decimalLongitude: -116.467933333333; geodeticDatum: WGS84; **Identification:** identifiedBy: Les Watling, Adrian Glover, Helena Wiklund, Thomas Dahlgren, Diva Amon; dateIdentified: 2016-03-01; identificationRemarks: identified by DNA and morphology; **Event:** samplingProtocol: Remotely Operated Vehicle; eventDate: 2013-10-17; eventTime: 19:46; habitat: Abyssal plain; fieldNumber: RV05; **Record Level:** language: en; institutionCode: NHMUK; collectionCode: ZOO; datasetName: ABYSSLINE; basisOfRecord: PreservedSpecimen
- **Type status:** Other material. **Occurrence:** catalogNumber: d24a6561-7c54-4607-89cd-6ddaaa557ca9; recordNumber: NHM_333; recordedBy: Adrian Glover, Helena Wiklund, Thomas Dahlgren, Maggie Georgieva; individualCount: 1; preparations: tissue voucher stored in 80% non-denatured ethanol aqueous solution and DNA voucher stored in elution buffer; otherCatalogNumbers: 5594613; associatedSequences: http://ncbi.nlm.nih.gov/nucleotide/KX384622 | KX384635; **Taxon:** taxonConceptID: Mopseinae sp. (NHM_330); scientificName: Mopseinae; kingdom: Animalia; phylum: Cnidaria; class: Anthozoa; order: Alcyonacea; family: Isididae; scientificNameAuthorship: Lamouroux, 1812; **Location:** waterBody: Pacific; stateProvince: Clarion Clipperton Zone; locality: UK Seabed Resources Ltd exploration claim UK-1; verbatimLocality: UK-1 Stratum A; maximumDepthInMeters: 4075; locationRemarks: RV Melville Cruise MV1313; decimalLatitude: 13.76085; decimalLongitude: -116.4653; geodeticDatum: WGS84; **Identification:** identifiedBy: Les Watling, Adrian Glover, Helena Wiklund, Thomas Dahlgren, Diva Amon; dateIdentified: 2016-03-01; identificationRemarks: identified by DNA and morphology; **Event:** samplingProtocol: Remotely Operated Vehicle; eventDate: 2013-10-17; eventTime: 19:06; habitat: Abyssal plain; fieldNumber: RV05; **Record Level:** language: en; institutionCode: NHMUK; collectionCode: ZOO; datasetName: ABYSSLINE; basisOfRecord: PreservedSpecimen
- **Type status:** Other material. **Occurrence:** catalogNumber: e54932dc-d64b-431f-8e55-fa93504e2bb6; recordNumber: NHM_376; recordedBy: Adrian Glover, Helena Wiklund, Thomas Dahlgren, Maggie Georgieva; individualCount: 1; preparations: tissue voucher stored in 80% non-denatured ethanol aqueous solution and DNA voucher stored in elution buffer; otherCatalogNumbers: 5594645; associatedSequences: http://ncbi.nlm.nih.gov/nucleotide/KX384636; **Taxon:** taxonConceptID: Mopseinae sp. (NHM_330); scientificName: Mopseinae; kingdom: Animalia; phylum: Cnidaria; class: Anthozoa; order: Alcyonacea; family: Isididae; scientificNameAuthorship: Lamouroux, 1812; **Location:** waterBody: Pacific; stateProvince: Clarion Clipperton Zone; locality: UK Seabed Resources Ltd exploration claim UK-1; verbatimLocality: UK-1 Stratum A; maximumDepthInMeters: 4182; locationRemarks: RV Melville Cruise MV1313; decimalLatitude: 13.933066666667; decimalLongitude: -116.71628333333; geodeticDatum: WGS84; **Identification:** identifiedBy: Les Watling, Adrian Glover, Helena Wiklund, Thomas Dahlgren, Diva Amon; dateIdentified: 2016-03-01; identificationRemarks: identified by DNA and morphology; **Event:** samplingProtocol: Brenke Epibenthic Sledge; eventDate: 2013-10-19; eventTime: 12:16; habitat: Abyssal plain; fieldNumber: EB05; fieldNotes: Collected from epi net (on the epibenthic sledge); **Record Level:** language: en; institutionCode: NHMUK; collectionCode: ZOO; datasetName: ABYSSLINE; basisOfRecord: PreservedSpecimen

#### Description

**Figure 8.**
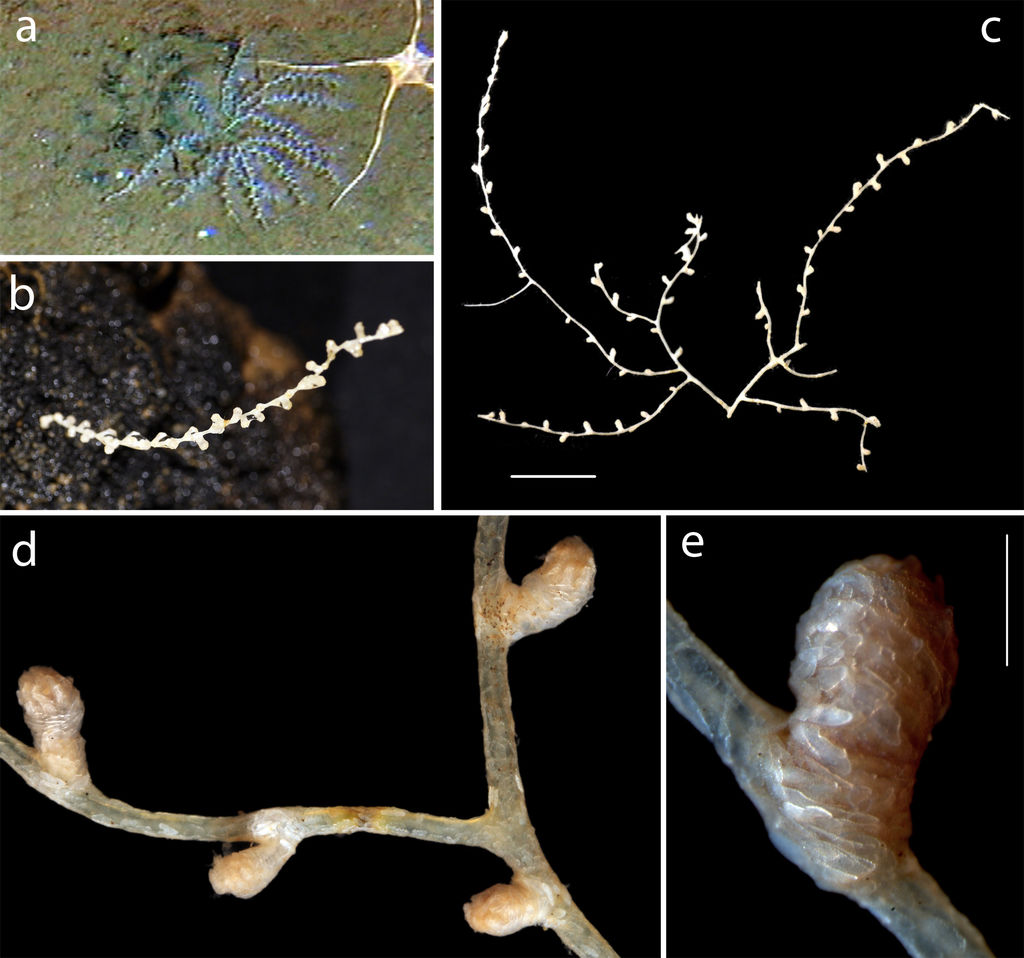
Mopseinae sp. 'NHM_330'. (a) Specimen NHM_330 attached to nodule imaged by Remotely Operated Vehicle in situ. (b) Specimen NHM_002. (c) Specimen NHM_330 imaged alive on ship. (d) Detail of NHM_330. (e) Detail of polyp, specimen NHM_330. Scale bars (c) 1 cm, (e) 500 µm. Image attribution (a) Diva Amon and Craig R Smith, (b-e) Glover, Dahlgren & Wiklund.

Branched or unbranched colonies attached to nodules. Identified to family based on general morphology (Fig. 8).

Genetic data for this taxon with new GenBank accession numbers are provided in Table 2​.

#### Diagnosis

No genetic matches on Genbank and does not cluster with other Isididae species available at Genbank but occupy a position ancestral to most other sequenced taxa in suborder Calcaxonia (order Alcyonacea) (Fig. 5). Based on morphology this taxon is here referred to the subfamily Mopseinae Gray, 1870. The genus is indeterminate at this time but morphologically resembles *Primnoisis* (Phil Alderslade *pers com*).

### Keratoisidinae sp. 'NHM_417'

#### Materials

- **Type status:** Other material. **Occurrence:** catalogNumber: d260bc48-6a67-4fb4-b504-863b6d174da0; recordNumber: NHM_417; recordedBy: Adrian Glover, Helena Wiklund, Thomas Dahlgren, Maggie Georgieva; individualCount: 1; preparations: tissue voucher stored in 80% non-denatured ethanol aqueous solution and DNA voucher stored in elution buffer; otherCatalogNumbers: 5594676; associatedSequences: http://ncbi.nlm.nih.gov/nucleotide/KX384623 | KX384637; **Taxon:** taxonConceptID: Keratoisidinae sp. (NHM_417); scientificName: Keratoisidinae; kingdom: Animalia; phylum: Cnidaria; class: Anthozoa; order: Alcyonacea; family: Isididae; scientificNameAuthorship: Lamouroux, 1812; **Location:** waterBody: Pacific; stateProvince: Clarion Clipperton Zone; locality: UK Seabed Resources Ltd exploration claim UK-1; verbatimLocality: UK-1 Stratum A; maximumDepthInMeters: 4026; locationRemarks: RV Melville Cruise MV1313; decimalLatitude: 13.8645666666667; decimalLongitude: -116.548233333333; geodeticDatum: WGS84; **Identification:** identifiedBy: Les Watling, Adrian Glover, Helena Wiklund, Thomas Dahlgren, Diva Amon; dateIdentified: 2016-03-01; identificationRemarks: identified by DNA and morphology; **Event:** samplingProtocol: Remotely Operated Vehicle; eventDate: 2013-10-20; eventTime: 11:05; habitat: Abyssal plain; fieldNumber: RV06; **Record Level:** language: en; institutionCode: NHMUK; collectionCode: ZOO; datasetName: ABYSSLINE; basisOfRecord: PreservedSpecimen

#### Description

**Figure 9.**
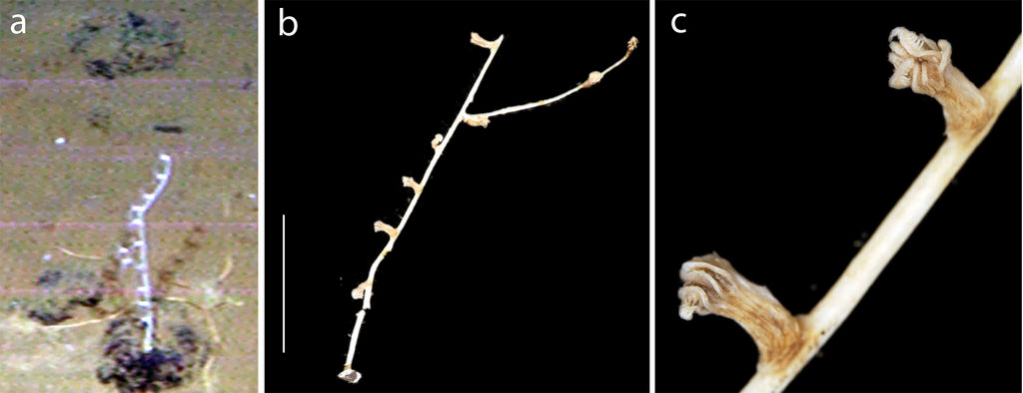
Keratoisidinae sp. 'NHM_417'. (a) Specimen imaged in situ by Remotely Operated Vehicle. Entire specimen. (b) Specimen after recovery. (c) Detail of polyps. Scale bar 3 cm. Image attribution (a) Diva Amon and Craig R Smith, (b-c) Glover, Dahlgren & Wiklund.

Branched colony attached to nodule. Identified to family based on general morphology (Fig. 9).

Genetic data for this taxon with new GenBank accession numbers are provided in Table 2.

#### Diagnosis

The specimen superficially resembles *Bathygorgia profunda* Wright (1885) first described in the Narrative (Part 2) of the HMS Challenger voyage and more fully described in Wright and Studer (1889) where a type locality from station 241 between Yokohama and Hawaii (depth 4206m) is indicated. Morphologically-similar specimens based on AUV or ROV image data is also referred to this species in the CCZ megafauna atlas (International Seabed Authority 2016). However, this specimen is distinguished by the presence of large needle sclerites (Fig. 9) that extend between the bases of the tentacles (which are not present in *Bathygorgia*), and the body sclerites being predominantly small needles rather than the disorganised rods that typify *Bathygorgia*. The present specimen belongs to a new genus and species (Watling et al. in prep). For a recent discussion on Keratoisid corals, see Dueñas et al. (2014) , Lapointe and Watling (2015) and Moore et al. 2016.

No genetic matches at Genbank but forms a cluster with *Keratoisis* and *Orstomisis* sequences published (Fig. 5).

### Hormathiidae sp. 'NHM_416'

#### Materials

- **Type status:** Other material. **Occurrence:** catalogNumber: bad617b4-5dff-4c9d-8ea8-c04ef29b0313; recordNumber: NHM_416; recordedBy: Adrian Glover, Helena Wiklund, Thomas Dahlgren, Maggie Georgieva; individualCount: 1; preparations: tissue voucher stored in 80% non-denatured ethanol aqueous solution and DNA voucher stored in elution buffer; otherCatalogNumbers: 5594675; associatedSequences: http://ncbi.nlm.nih.gov/nucleotide/KX384620; **Taxon:** taxonConceptID: Hormathiidae sp. (NHM_416); scientificName: Hormathiidae; kingdom: Animalia; phylum: Cnidaria; class: Anthozoa; order: Actiniaria; family: Hormathiidae; scientificNameAuthorship: Carlgren, 1932; **Location:** waterBody: Pacific; stateProvince: Clarion Clipperton Zone; locality: UK Seabed Resources Ltd exploration claim UK-1; verbatimLocality: UK-1 Stratum A; maximumDepthInMeters: 4025; locationRemarks: RV Melville Cruise MV1313; decimalLatitude: 13.8645283333333; decimalLongitude: -116.548211666667; geodeticDatum: WGS84; **Identification:** identifiedBy: Estefania Rodriguez, Adrian Glover, Helena Wiklund, Thomas Dahlgren, Diva Amon; dateIdentified: 2016-03-01; identificationRemarks: identified by DNA and morphology; **Event:** samplingProtocol: Remotely Operated Vehicle; eventDate: 2013-10-20; eventTime: 22:54; habitat: Abyssal plain; fieldNumber: RV06; **Record Level:** language: en; institutionCode: NHMUK; collectionCode: ZOO; datasetName: ABYSSLINE; basisOfRecord: PreservedSpecimen

#### Description

**Figure 10.**
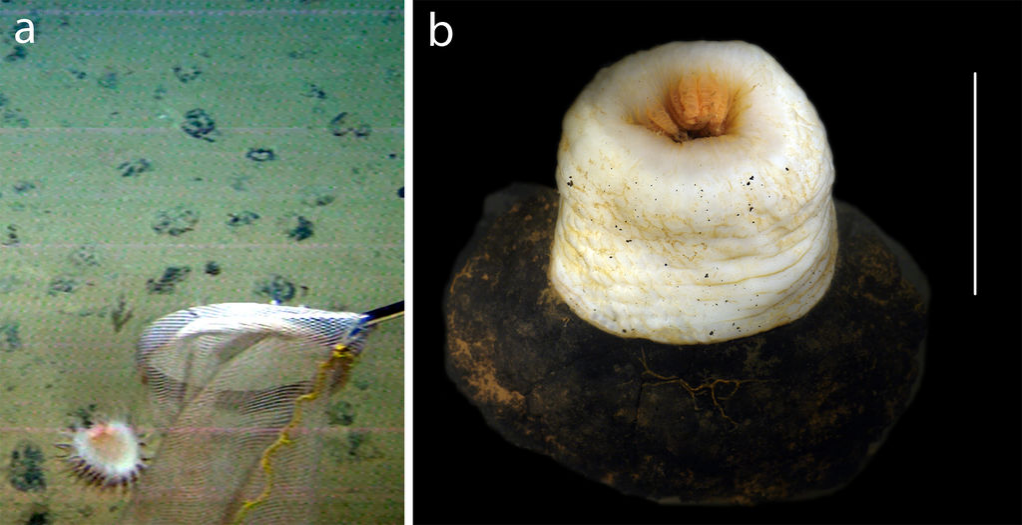
Hormathiidae sp. NHM_416. Scale bar 5 cm. Image attribution (a) Diva Amon & Craig R Smith, (b) Glover, Dahlgren & Wiklund.

Relatively large anemone attached to nodule. Column pale, white, with verrucae arranged in rows. No pronounced limbus. Morphology and colour of tentacles not observed (Fig. 10).

Genetic data for this taxon with new GenBank accession numbers are provided in Table 2.

#### Diagnosis

Assigned here to the family Hormathiidae Carlgren (1932) based on morphology, and most similar to species in the genus *Phelliactis* Simon (1892) (Estefania Rodriguez pers. com.). Attempts to collect a full suite of genetic marker data was unsuccessful, and limited to the 18S gene only. A phylogenetic analysis of the 18S gene including hexacoral taxa available at Genbank placed this specimen with low support as sister to a sequence assigned to *Capnea georgiana* (Carlgren 1927). The result is most probably an artefact due to the restricted dataset and homoplasy in the 18S gene. Previous analyses has suggested limitation of this gene alone for species barcoding and genus level phylogenetic reconstruction in hexacorals (Rodríguez et al. 2014).

### Abylopsis eschscholtzii

(Huxley, 1859)

#### Materials

- **Type status:** Other material. **Occurrence:** catalogNumber: 8cab45b5-4868-4d7a-a2a1-92f8b9ab3649; recordNumber: NHM_477; recordedBy: Adrian Glover, Helena Wiklund, Thomas Dahlgren, Maggie Georgieva; individualCount: 1; preparations: tissue voucher stored in 80% non-denatured ethanol aqueous solution and DNA voucher stored in elution buffer; otherCatalogNumbers: 5595047; associatedSequences: http://ncbi.nlm.nih.gov/nucleotide/KX384609 | KX384617; **Taxon:** taxonConceptID: Abylopsis eschscholtzii; scientificName: Abylopsis eschscholtzii; kingdom: Animalia; phylum: Cnidaria; class: Hydrozoa; order: Siphonophorae; family: Abylidae; genus: Abylopsis; specificEpithet: eschscholtzii; scientificNameAuthorship: Huxley, 1859; **Location:** waterBody: Pacific; stateProvince: Clarion Clipperton Zone; locality: UK Seabed Resources Ltd exploration claim UK-1; verbatimLocality: UK-1 Stratum A; maximumDepthInMeters: 4160; locationRemarks: RV Melville Cruise MV1313; decimalLatitude: 13.726616666667; decimalLongitude: -116.67; geodeticDatum: WGS84; **Identification:** identifiedBy: Adrian Glover, Helena Wiklund, Thomas Dahlgren; dateIdentified: 2016-03-01; identificationRemarks: identified by DNA and morphology; **Event:** samplingProtocol: USNEL Box Core; eventDate: 2013-10-22; eventTime: 07:25; habitat: Abyssal plain; fieldNumber: BC14; fieldNotes: Collected from 0-2 cm layer of box core using a 300 micron sieve; **Record Level:** language: en; institutionCode: NHMUK; collectionCode: ZOO; datasetName: ABYSSLINE; basisOfRecord: PreservedSpecimen

#### Description

**Figure 11.**
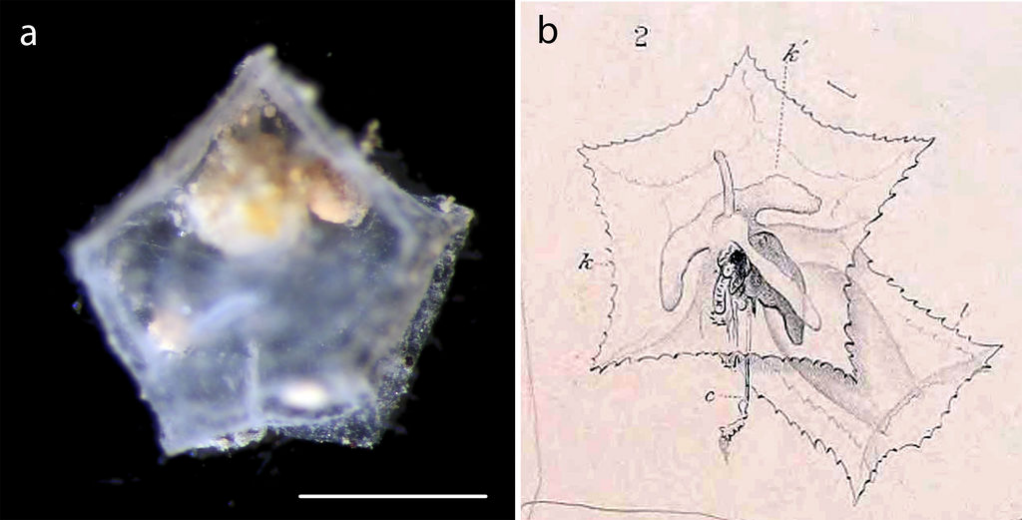
*Abylopsis eschscholtzii* (Huxley, 1859). (a) Specimen NHM_477. (b) Drawing from original description by Huxley 1859. Scale bar 1 mm. Image attribution (a) Glover, Dahlgren & Wiklund 2016.

Pentagonal, transparent disk found in a boxcore sample, possibly from the water column (Fig. 11). The animal cavity contained sediment.

Genetic data for this taxon with new GenBank accession numbers are provided in Table 2.

#### Diagnosis

The CO1 data suggest that the specimen is identical to *Abylopsis eschscholtzii* (Huxley, 1859) (Genbank Accession # GQ119937). No type locality was assigned to the name in the species description. The original description state that the species occurred in 'all the seas that was traversed' (Huxley 1859).

### Corymorphidae sp. 'NHM_398'

#### Materials

- **Type status:** Other material. **Occurrence:** catalogNumber: dc50e246-ae77-401d-9d57-8f0c8df2b07d; recordNumber: NHM_398; recordedBy: Adrian Glover, Helena Wiklund, Thomas Dahlgren, Maggie Georgieva; individualCount: 1; preparations: tissue voucher stored in 80% non-denatured ethanol aqueous solution and DNA voucher stored in elution buffer; otherCatalogNumbers: 5594663; associatedSequences: http://ncbi.nlm.nih.gov/nucleotide/KX384628; **Taxon:** taxonConceptID: Corymorphidae sp. (NHM_398); scientificName: Corymorphidae; kingdom: Animalia; phylum: Cnidaria; class: Hydrozoa ; order: Anthoathecata; family: Corymorphidae; scientificNameAuthorship: Allman,1872; **Location:** waterBody: Pacific; stateProvince: Clarion Clipperton Zone; locality: UK Seabed Resources Ltd exploration claim UK-1; verbatimLocality: UK-1 Stratum A; maximumDepthInMeters: 4500; locationRemarks: RV Melville Cruise MV1313; decimalLatitude: 13.863283333333; decimalLongitude: -116.54885; geodeticDatum: WGS84; **Identification:** identifiedBy: Adrian Glover, Helena Wiklund, Thomas Dahlgren, Diva Amon; dateIdentified: 2016-03-01; identificationRemarks: identified by DNA and morphology; **Event:** samplingProtocol: USNEL Box Core; eventDate: 2013-10-20; eventTime: 03:39; habitat: Abyssal plain; fieldNumber: BC12; fieldNotes: Collected from 0-2 cm layer of box core using a 300 micron sieve; **Record Level:** language: en; institutionCode: NHMUK; collectionCode: ZOO; datasetName: ABYSSLINE; basisOfRecord: PreservedSpecimen

#### Description

**Figure 12.**
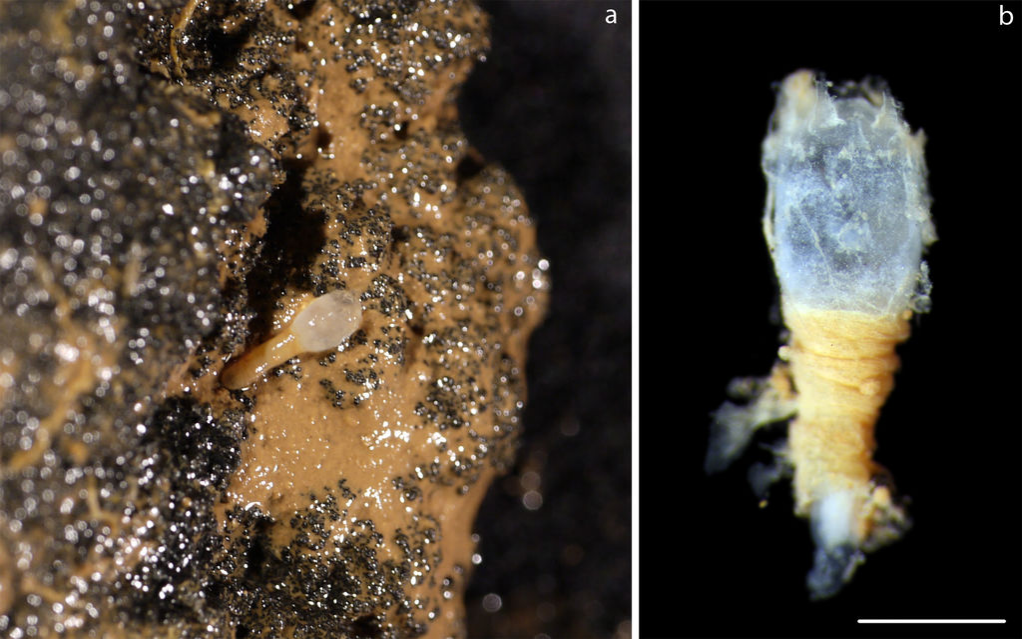
Corymorphidae sp. NHM_398. (a) Specimen NHM_398 attached to nodule, (b) Detail, same specimen. Scale bar 1,5 mm. Image attribution Glover, Dahlgren & Wiklund 2016.

Small bulb-like corymorph hydrozoan found attached to a nodule with the crown apparently retracted (Fig. 12).

Genetic data for this taxa with new Genbank accession numbers are provided in Table 2.

#### Diagnosis

Phylogenetic analyses based on gene data suggest that this hydrozoan fall within the family Corymorphidae (tree not showed).

### Siphonophorae sp. 'NHM_339'

#### Materials

- **Type status:** Other material. **Occurrence:** catalogNumber: 42ffb986-0c1c-45ed-be56-7b9512ed746f; recordNumber: NHM_339; recordedBy: Adrian Glover, Helena Wiklund, Thomas Dahlgren, Maggie Georgieva; individualCount: 1; preparations: tissue voucher stored in 80% non-denatured ethanol aqueous solution and DNA voucher stored in elution buffer; otherCatalogNumbers: 5594616; associatedSequences: http://ncbi.nlm.nih.gov/nucleotide/KX384615 | KX384625; **Taxon:** taxonConceptID: Siphonophorae sp. (NHM_339); scientificName: Siphonophorae; kingdom: Animalia; phylum: Cnidaria; class: Hydrozoa ; order: Siphonophorae; scientificNameAuthorship: Eschscholtz, 1829; **Location:** waterBody: Pacific; stateProvince: Clarion Clipperton Zone; locality: UK Seabed Resources Ltd exploration claim UK-1; verbatimLocality: UK-1 Stratum A; maximumDepthInMeters: 4111; locationRemarks: RV Melville Cruise MV1313; decimalLatitude: 13.761666666667; decimalLongitude: -116.46033333333; geodeticDatum: WGS84; **Identification:** identifiedBy: Adrian Glover, Helena Wiklund, Thomas Dahlgren; dateIdentified: 2016-03-01; identificationRemarks: identified by DNA and morphology; **Event:** samplingProtocol: Bowers & Connelly Megacore; eventDate: 2013-10-18; eventTime: 15:54; habitat: pelagic; fieldNumber: MC08; **Record Level:** language: en; institutionCode: NHMUK; collectionCode: ZOO; datasetName: ABYSSLINE; basisOfRecord: PreservedSpecimen

#### Description

**Figure 13.**
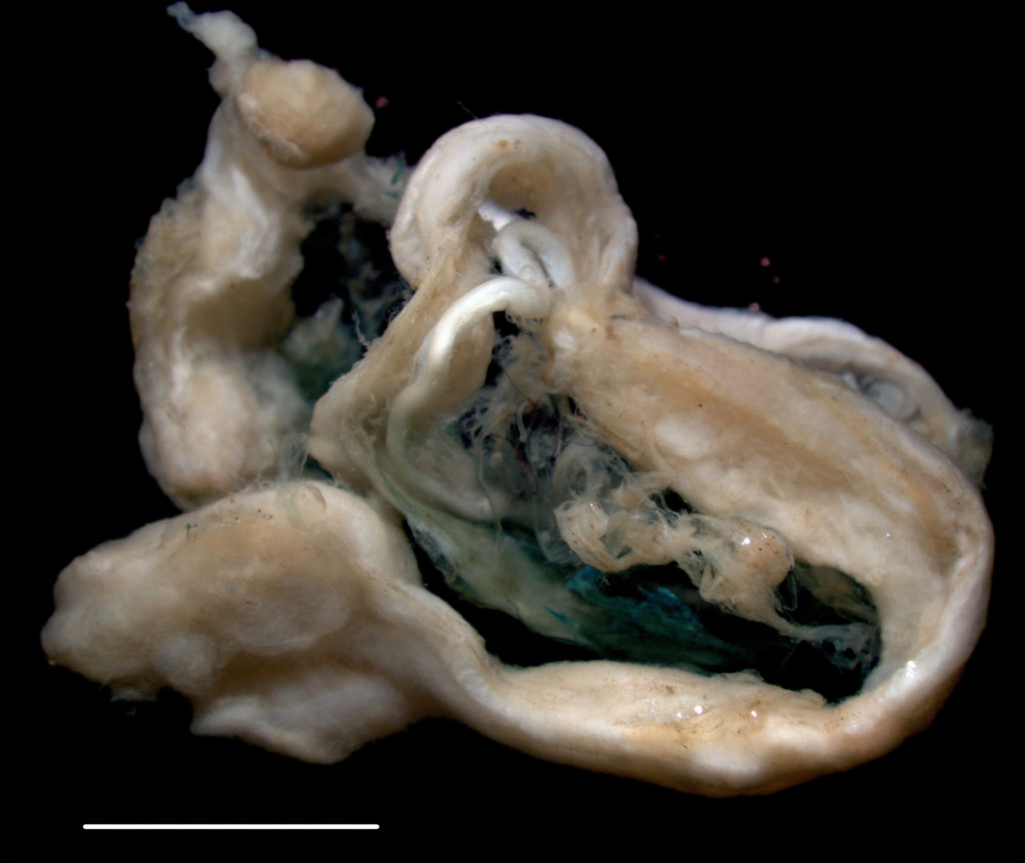
Siphonophorae sp. NHM_339. Detached tentacle found on pinger. Scale bar 5 mm. Image attribution Glover, Dahlgren & Wiklund 2016.

Tentacle from pelagic jellyfish found on pinger (Fig. 13).

Genetic data for this taxon with new GenBank accession numbers are provided in Table 2.

#### Diagnosis

No genetic matches at Genbank but in a COI tree (not shown) it is sister to *Nectopyramis natans* although with quite low support, while the 18S sequence BLAST search is nearest to *Rhizophysa eysenhardti*.

### Stephanomia amphytridis

Lesueur & Petit, 1807

#### Materials

- **Type status:** Other material. **Occurrence:** catalogNumber: a1573f3a-6acf-46b9-b43e-db3a9ac90d2b; recordNumber: NHM_249; recordedBy: Adrian Glover, Helena Wiklund, Thomas Dahlgren, Maggie Georgieva; individualCount: 1; preparations: tissue voucher stored in 80% non-denatured ethanol aqueous solution and DNA voucher stored in elution buffer; otherCatalogNumbers: 5594546; associatedSequences: http://ncbi.nlm.nih.gov/nucleotide/KX384616; **Taxon:** taxonConceptID: Stephanomia amphytridis; scientificName: Stephanomia amphytridis; kingdom: Animalia; phylum: Cnidaria; class: Hydrozoa; order: Siphonophorae; family: Stephanomiidae; genus: Stephanomia; specificEpithet: amphytridis; scientificNameAuthorship: Lesueur & Petit, 1807; **Location:** waterBody: Pacific; stateProvince: Clarion Clipperton Zone; locality: UK Seabed Resources Ltd exploration claim UK-1; verbatimLocality: UK-1 Stratum A; maximumDepthInMeters: 4076; locationRemarks: RV Melville Cruise MV1313; decimalLatitude: 13.811683333333; decimalLongitude: -116.71001666667; geodeticDatum: WGS84; **Identification:** identifiedBy: Adrian Glover, Helena Wiklund, Thomas Dahlgren; dateIdentified: 2016-03-01; identificationRemarks: identified by DNA and morphology; **Event:** samplingProtocol: Bowers & Connelly Megacore; eventDate: 2013-10-16; eventTime: 19:50; habitat: pelagic; fieldNumber: MC06; **Record Level:** language: en; institutionCode: NHMUK; collectionCode: ZOO; datasetName: ABYSSLINE; basisOfRecord: PreservedSpecimen

#### Description

Fragment of specimen stuck on megacore at retrieval. No image.

Genetic data for this taxon with new GenBank accession numbers are provided in Table 2.

#### Diagnosis

The CO1 data suggest that the specimen is identical to *Stephanomia amphytridis* Lesueur and Petit (1807) (Genbank Accession # GQ120047).

### Nausithoe sp. 'NHM_353'

#### Materials

- **Type status:** Other material. **Occurrence:** catalogNumber: 86032849-ddd2-4690-b5e3-a3d1a7d09d3d; recordNumber: NHM_353; recordedBy: Adrian Glover, Helena Wiklund, Thomas Dahlgren, Maggie Georgieva; individualCount: 2; preparations: tissue voucher stored in 80% non-denatured ethanol aqueous solution and DNA voucher stored in elution buffer; otherCatalogNumbers: 5595043; associatedSequences: http://ncbi.nlm.nih.gov/nucleotide/KX384614 | KX384624; **Taxon:** taxonConceptID: Nausithoe sp. (NHM_353); scientificName: Nausithoe; kingdom: Animalia; phylum: Cnidaria; class: Scyphozoa; order: Coronatae; family: Nausithoidae; genus: Nausithoe; scientificNameAuthorship: Kölliker, 1853; **Location:** waterBody: Pacific; stateProvince: Clarion Clipperton Zone; locality: UK Seabed Resources Ltd exploration claim UK-1; verbatimLocality: UK-1 Stratum A; maximumDepthInMeters: 4150; locationRemarks: RV Melville Cruise MV1313; decimalLatitude: 13.888333333333; decimalLongitude: -116.68998333333; geodeticDatum: WGS84; **Identification:** identifiedBy: Andy Gooday, Adrian Glover, Helena Wiklund, Thomas Dahlgren; dateIdentified: 2016-03-01; identificationRemarks: identified by DNA and morphology; **Event:** samplingProtocol: USNEL Box Core; eventDate: 2013-10-19; eventTime: 02:25; habitat: Abyssal plain; fieldNumber: BC11; fieldNotes: Collected from 0-2 cm layer of box core using a 300 micron sieve; **Record Level:** language: en; institutionCode: NHMUK; collectionCode: ZOO; datasetName: ABYSSLINE; basisOfRecord: PreservedSpecimen

#### Description

**Figure 14.**
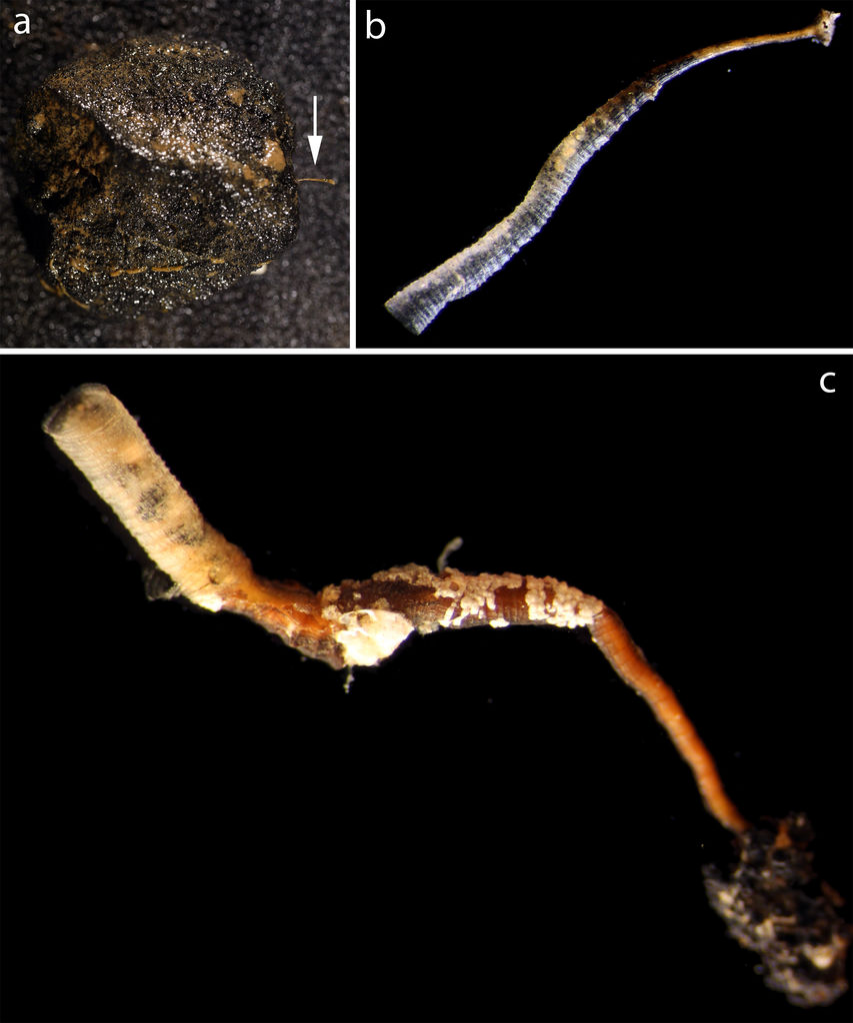
*Nausithoe* sp. NHM_353. (a) Specimen NHM_321 attached to nodule (arrow). Image attribution Glover, Dahlgren & Wiklund 2016.

Specimens attached to nodule. Seemingly empty tubes with transverse rings and longitudinal striations (Fig. 14).

Genetic data for this taxon (NHM_353) with new GenBank accession numbers are provided in Table 2.

#### Diagnosis

ID based on Genbank BLAST place the material close to *Nausithoe atlantica* Broch (1914). The tubes are morphologically similar to *Atorella sibogae* (Leloup 1937) (top fig. p 119 in Morandini and Jarms (2005). The species was previously assigned to *Stephanoscyphus* based on general polyp morphology (Morandini and Jarms 2005). Type locality of *Atorella sibogae* is Malayan archipelago at 794 m depth. The specimens NHM_083 and NHM_321 failed to produce any DNA data and the identification is therefor provisional.

## Discussion

After more than three decades of explorations focused on the CCZ, faunal records from the area (e.g. in the OBIS or GBIF databases) remain scarce. For the entire 6 million sq km area, there are no more than 220 records belonging to 52 taxa. Limiting the search for the same area to benthic records of Cnidaria yielded seven records of five taxa. In this study we report 18 records for 10 taxa, most of which benthic, that is more than doubling the known data for the region from our relatively small sample set. There were only two matches in Genbank for any of our cnidarian sequences. They were for the two pelagic hydrozoans *Abylopsis eschscholtzii* and *Stephanomia amphytridis*, two species with supposedly cosmopolitan distributions but previously not reported from the CCZ. A third pelagic species (Siphonophorae sp.) did not match any previously sequenced taxon available on Genbank.

Deep-sea corals are, in general, considered as vulnerable to anthropogenic stressors such as ocean acidification (Orr et al. 2005) and increased turbidity from activities such as trawling or oil and gas extraction (Roberts 2002). Changes in species composition and abundance of the diverse, and to a considerable extent, novel coral fauna of the CCZ, may become an important indicator of environmental impact from future mining activities. Our data show that DNA sampling of this fauna in conjuction with detailed morphological examination is required to properly assess CCZ coral diversity. We hope that the rapid publication of this and similar taxonomic data from other groups (Glover et al. 2016) will help ongoing and future efforts to build a taxonomic baseline of the CCZ suited to future impact assessments and environmental monitoring.

## Supplementary Material

Supplementary material 1 Taxa table Data type: Genbank accession numbers
Brief description: List of taxa downloaded from GenBank that are included in the phylogenetic analysis of Octocorallia, with their Genbank accession numbers. Accession numbers for taxa sequenced in this study are found in Table 2.
File: oo_86279.xlsx Dahlgren et al.

XML Treatment for Abyssoprimnoa gemina

XML Treatment for Calyptrophora persephone

XML Treatment for Mopseinae sp. 'NHM_330'

XML Treatment for Keratoisidinae sp. 'NHM_417'

XML Treatment for Hormathiidae sp. 'NHM_416'

XML Treatment for Abylopsis eschscholtzii

XML Treatment for Corymorphidae sp. 'NHM_398'

XML Treatment for Siphonophorae sp. 'NHM_339'

XML Treatment for Stephanomia amphytridis

XML Treatment for Nausithoe sp. 'NHM_353'

## Figures and Tables

**Table 1.** Primers used for PCR and sequencing of 18S, 16S and COI.

Primer	Sequence 5'-3'	Reference
18S		
18SA	AYCTGGTTGATCCTGCCAGT	Medlin et al. 1988
18SB	ACCTTGTTACGACTTTTACTTCCTC	Nygren and Sundberg 2003
620F	TAAAGYTGYTGCAGTTAAA	Nygren and Sundberg 2003
1324R	CGGCCATGCACCACC	Cohen et al. 1998
CO1		
LCO1490	GGTCAACAAATCATAAAGATATTGG	Folmer et al. 1994
HCO2198	TAAACTTCAGGGTGACCAAAAAATCA	Folmer et al. 1994
polyLCO	GAYTATWTTCAACAAATCATAAAGATATTGG	Carr et al. 2011
polyHCO	TAMACTTCWGGGTGACCAAARAATCA	Carr et al. 2011
16S		
16SbrH	CCGGTCTGAACTCAGATCACGT	Palumbi 1996
Ann16SF	GCGGTATCCTGACCGTRCWAAGGTA	Sjölin et al. 2005

**Table 2.** Taxon treatments presented in this paper. Includes Class, DNA Taxonomy ID (a species-level identification based on combined DNA and morphological evidence), GUID (Global Unique Identifier link to data record on http://data.nhm.ac.uk), ABYSSLINE Record number, NHM Accession number, NHM Molecular Collection Facility (MCF) sample ID number (NHMUK_MCF#) and NCBI GenBank accession number (Genbank#) for successfully sequenced genetic markers. Record numbers include both material archived at NHM (all DNA tissue samples held in the NHM MCF, plus morphological material including whole specimens for some taxa), University of Hawaii (AB01_CSXX) and National Museum of Natural History (USNM). GenBank numbers for data downloaded from GenBank for phylogenetic analysis are presented in Suppl. material 1​.

Class	DNA Taxonomy ID	GUID#	ABYSSLINE Record#	NHMUK_Acc#	NHMUK_MCF#	Genbank#
Anthozoa	*Abyssoprimnoa gemina*	6e976c27-c70c-434a-8be3-f6155e36567f	NHM_341 USNM 1268848 AB01_CS09	NHMUK 2016.1	0185546837 0185546838 0185546851	KX384618 KX384626
Anthozoa	*Calyptrophora persephone*	dc143dd0-a366-479a-9e23-ca28fe79761b	NHM_462 USNM 1268754 AB01_CS16	NHMUK 2016.2	0185546835 0185546836 0185546847	KX384619 KX384627
Anthozoa	Mopseinae sp. (NHM_330)	52fc66a5-d396-49be-a210-4ac58a164aec	NHM_002	NHMUK 2016.4	0185546839 0185546840 0185546862	KX384629
Anthozoa	Mopseinae sp. (NHM_330)	c1c12d85-274b-4ae5-bf60-72c82b70dc16	NHM_019	NHMUK 2016.5	0185546841 0185546863	KX384610 KX384621 KX384630
Anthozoa	Mopseinae sp. (NHM_330)	39fb53c7-b5f4-4e9a-a8da-756129250937	NHM_035	NHMUK 2016.6	0185546842 0185546864	KX384611
Anthozoa	Mopseinae sp. (NHM_330)	fb7d366b-dcd6-448e-8fe0-7625d811483d	NHM_136	NHMUK 2016.7	0185541733 0185546843 0185546865	KX384631
Anthozoa	Mopseinae sp. (NHM_330)	6102fb1a-c452-47a8-9a52-ca1159c06cb0	NHM_154	NHMUK 2016.8	0185546830 0185546866	KX384632
Anthozoa	Mopseinae sp. (NHM_330)	d73e2414-e2ad-4c07-9e12-0864ce6ef3c1	NHM_203	NHMUK 2016.9	0185546867	KX384612 KX384633
Anthozoa	Mopseinae sp. (NHM_330)	d5fdef19-8efd-4865-bc53-725568bac8df	NHM_330 AB01_CS07	NHMUK 2016.10	0185546828 0185546829 0185546854	KX384613 KX384634
Anthozoa	Mopseinae sp. (NHM_330)	d24a6561-7c54-4607-89cd-6ddaaa557ca9	NHM_333	NHMUK 2016.11	0185541760 0185546826 0185546827	KX384622 KX384635
Anthozoa	Mopseinae sp. (NHM_330)	e54932dc-d64b-431f-8e55-fa93504e2bb6	NHM_376	NHMUK 2016.12	0185546824 0185546825 0185546849	KX384636
Anthozoa	Keratoisidinae sp. (NHM_417)	d260bc48-6a67-4fb4-b504-863b6d174da0	NHM_417 AB01_CS14	NHMUK 2016.13	0185546822 0185546823 0185546848	KX384623 KX384637
Anthozoa	Hormathiidae sp. (NHM_416)	bad617b4-5dff-4c9d-8ea8-c04ef29b0313	NHM_416 AB01_CS13	NHMUK 2016.3	0185541761 0185546820 0185546821	KX384620
Hydrozoa	*Abylopsis eschscholtzii*	8cab45b5-4868-4d7a-a2a1-92f8b9ab3649	NHM_477	NHMUK 2016.14	0185546846	KX384609 KX384617
Hydrozoa	Corymorphidae sp. (NHM_398)	dc50e246-ae77-401d-9d57-8f0c8df2b07d	NHM_398	NHMUK 2016.15	0185546845	KX384628
Hydrozoa	Siphonophorae sp. (NHM_339)	42ffb986-0c1c-45ed-be56-7b9512ed746f	NHM_339	NHMUK 2016.16	0185546808 0185546809 0185546852	KX384615 KX384625
Hydrozoa	*Stephanomia amphytridis*	a1573f3a-6acf-46b9-b43e-db3a9ac90d2b	NHM_249	NHMUK 2016.17	0185546811 0185546812 0185546832	KX384616
Scyphozoa	*Nausithoe* sp. (NHM_353)	86032849-ddd2-4690-b5e3-a3d1a7d09d3d	NHM_353	NHMUK 2016.18	0185546810 0185546850	KX384614 KX384624
